# Gliomatosis Cerebri in the Brain of a Cat

**DOI:** 10.3390/vetsci3030013

**Published:** 2016-06-27

**Authors:** Stephanie Shrader, Serene Lai, Kelsey Cline, Rachel Moon

**Affiliations:** 1 Department of Pathobiology, College of Veterinary Medicine, Auburn University, Auburn, AL 36849, USA; 2 Department of Clinical Sciences, College of Veterinary Medicine, Auburn University, Auburn, AL 36849, USA; srl0014@tigermail.auburn.edu (S.L.); kac0050@tigermail.auburn.edu (K.C.); rsm0026@auburn.edu (R.M.)

**Keywords:** cat, gliomatosis cerebri, brain, T2 hyperintensity

## Abstract

An eight-year-old, neutered, female, long-haired cat was presented with a three-week history of progressive lethargy, unlocalized pain in the cervical and lumbar spine, and unwillingness to move. An MRI (magnetic resonance imaging) of the brain revealed poorly circumscribed regions of non-contrast-enhancing heterogeneous T2 hyperintensity within the ventral forebrain and midbrain. A mass effect and evidence of increased intracranial pressure, including transtentorial herniation of the midbrain and herniation of the cerebellar vermis through the foramen magnum, were also observed. Due to progressive clinical decline and MRI results, the cat was humanely euthanized. Gross examination of the brain confirmed caudal transtentorial and foramen magnum herniation. The ventral aspect of the forebrain, midbrain, and brainstem were soft and had loss of detail, but lacked a grossly discernible mass. Histopathological examination found a poorly delineated neoplastic mass composed of hyperchromatic cells with indistinct cytoplasm, ovoid to elongate or curved nuclei, and indistinct nucleoli. The cells lacked immunoreactivity for Olig2, GFAP, Iba1, CD3, and Pax5. Based on the cellular morphology, immunolabeling characteristics, and anatomical location, a diagnosis of gliomatosis cerebri was made. Although uncommon, gliomatosis cerebri should be considered as a differential diagnosis in cats with central nervous system disease.

## 1. Introduction

Gliomatosis cerebri (GC) is an uncommon glial neoplasm, first reported by Nevin [1]. Although it is characterized by neoplastic glial cells that infiltrate the neuroparenchyma, a grossly discernible mass is often lacking [2]. The 2007 World Health Organization (WHO) classification defines human GC as an astrocytic tumor [3]; however, scholars debate whether GC represents a separate entity or a subset of diffuse glioma [4]. Clonal analysis of gliomas has found that GC begins as an oligoclonal process or may result from collision gliomas. This differs from the monoclonality typically observed in low grade and malignant gliomas [5]. In veterinary medicine, GC has been classified as either a miscellaneous type of tumor [6] or as a neuroepithelial neoplasm of unknown origin [7].

## 2. Case History

An eight-year-old, neutered, female, long-haired cat was presented to the Wilford & Kate Bailey Small Animal Teaching Hospital at Auburn University’s College of Veterinary Medicine (AUCVM) with a three-week history of progressive lethargy, unlocalized pain in the cervical and lumbar spine, and unwillingness to move. Two weeks prior to presentation at AUCVM, the cat was presented to a local emergency veterinary clinic and reportedly diagnosed with diskospondylitis (based on thoracic radiographs). She was treated with steroids (prednisone, dosage unknown) and butorphanol (0.09 mg/kg PO as needed) resulting in improvement of clinical signs. Over the next couple weeks, the cat experienced a recurrence of cervical pain and a progressive decline in mentation. She presented to the referring veterinarian and was treated with prednisone (increased from 0.9 mg/kg every 48 to 24 h; last dose three days prior to AUCVM presentation), Depo-Medrol (dosage unknown; last dose one day prior to AUCVM presentation), and intermittent doses of butorphanol (0.09 mg/kg PO as needed; last dose two days prior to AUCVM presentation). The cat’s mentation declined despite treatment and on the morning of presentation to AUCVM, she experienced a tonic seizure that lasted for 20 s. The cat was taken to the referring veterinarian who administered midazolam (dosage unknown) and referred the cat to AUCVM.

On presentation to AUCVM, the cat had a body weight of 5.7 kg, a body condition score of 4/5, and a rectal temperature of 102.3°F. A neurologic examination found that the cat was dull but responsive, with mild proprioceptive ataxia in all four limbs. Cranial nerve examination and segmental reflexes were normal. Postural reactions were considered to be normal, with the exception of possible delayed hopping in the right thoracic limb. There was hyperesthesia associated with palpation of the cranial cervical vertebrae and auditory bullae; however, it was difficult to determine whether this response was behavioral or pain-related. Bloodwork and radiographs, performed by the referring veterinarian, were reviewed. Hematological and serum biochemical parameters were within reference intervals; spinal radiographs showed spondylosis deformans at L3-L4, but no evidence of diskospondylitis. 

Based on clinical findings, a forebrain lesion was considered most likely and magnetic resonance imaging (MRI) of the brain was performed using a 1.5 T scanner and a human knee coil (Infinion: Philips Medical Systems, Andover, MA, USA). Sagittal images were obtained with T2-weighting (T2) (TR 4000 TE 118.3, 3 mm). Transverse images included T2 (TR 4000, TE 145.6, 3 mm), proton density (TR 4000, TE 15, 3 mm), fluid attenuated inversion recovery (FLAIR) (TR 6000, TE 111.6, 3 mm), gradient recalled echo (GRE) (TR 1188, TE 23.5, 3 mm), and T1-weighting (T1) (TR 400, TE 11.5, 3 mm). Post-intravenous contrast T1 images were obtained in all three standard planes approximately 5 min after manual intravenous injection of gadolinium-based contrast (transverse, sagittal and dorsal: TR 400, TE 11.5, 3 mm) (0.11 mmol/kg body weight, gadoteridol (Prohance; Bracco Diagnostic, Inc., Township, NJ, USA)). 

**Figure 1:**
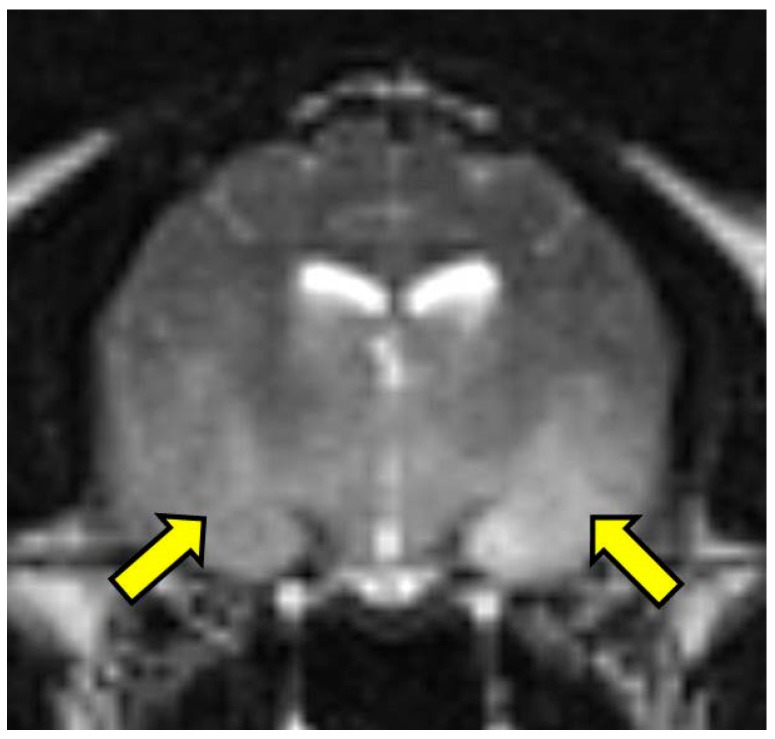
T2 weighted transverse image at the level of the piriform lobe showing the ill-defined, ventrally distributed T2-weighted hyperintensity affecting the piriform lobes, temporal lobes, hypothalamus, and portions of the caudal colliculus and internal capsule (arrows). MRI scan.

**Figure 2:**
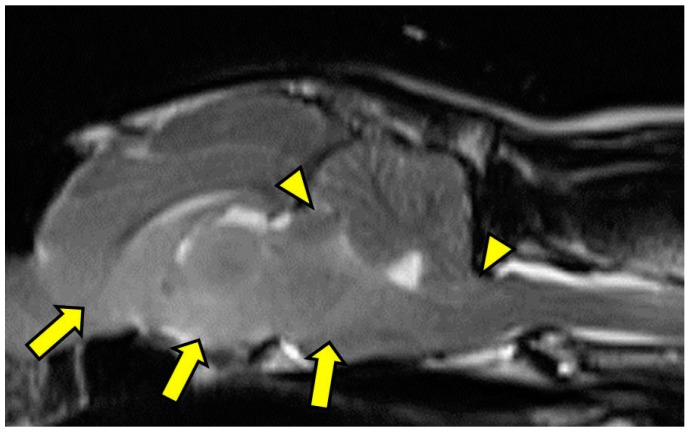
T2-weighted midline sagittal image showing caudal transtentorial herniation of the midbrain through the osseous tentorium and caudal herniation of the cerebellum through the foramen magnum (arrowheads). Additionally, there is an ill-defined, mildly hyperintense mass effect in the ventral aspect of forebrain including the caudal nucleus of the thalamus, interthalamic adhesion, colliculi, and midbrain (arrows). MRI scan.

On MRI scanning of the brain, an intra-axial, diffuse, non-contrast-enhancing, T2-weighted, hyperintense mass effect extending through the ventral forebrain (including the piriform and temporal lobes, thalamus, crus cerebri, and colliculi) and midbrain (Figure 1) was found. The caudal aspects of the cerebellum were compressed against the occipital bone. There was also caudal transtentorial herniation of the midbrain and herniation of the cerebellar vermis through the foramen magnum (Figure 2). A linear, T2 hyperintense area was present in the dorsal aspect of the C2-C5 spinal cord; transverse images of this area showed dilation of the central canal, T2 hyperintensity, and non-contrast-enhancing T1 hypointensity in the dorsal spinal cord, most consistent with syrinx formation. Based on clinical findings and MRI results, differential diagnoses included multicentric neoplasia or an infectious etiology such as feline infectious peritonitis (FIP), feline immunodeficiency virus (FIV), feline leukemia virus (FeLV), or cryptococcosis. Due to poor prognosis and rapid clinical decline, the cat was humanely euthanized. 

**Figure 3:**
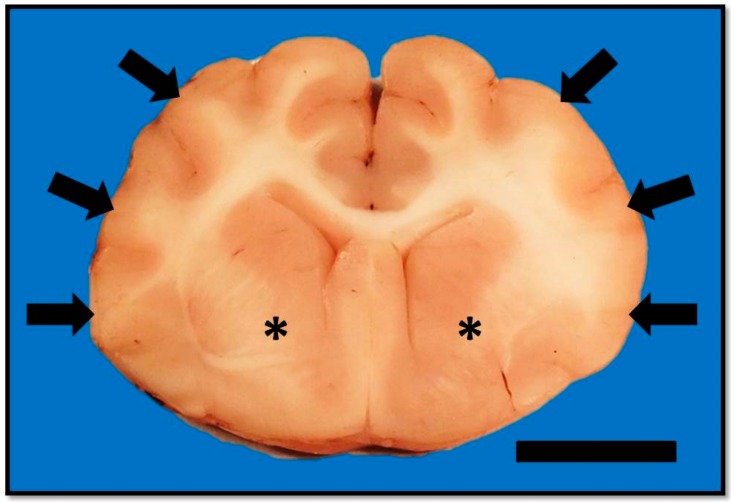
Transverse section of formalin-fixed brain at the level of the septal nuclei. There is mild to moderate flattening of the suprasylvian, ectosylvian, and sylvian gyri (arrows) with narrowing of the associated sulci. The ventral aspects of the corpus striatum (astrices) have bilateral but asymmetrical loss of detail. Bar = 1 cm.

On post-mortem examination, changes were confined to the central nervous system. Gross examination of the brain confirmed compression of the caudal cerebellum, caudal transtentorial herniation of the midbrain, and herniation of the cerebellar vermis. There was mild to moderate flattening of the straight, suprasylvian, ectosylvian, sylvian, dentate, and occipital gyri with narrowing of the rostral rhinal, lateral rhinal, suprasylvian, and ectosylvian sulci. On the cut surface, the corpus striatum, thalamus, and hippocampus were soft and had loss of detail, but lacked a grossly discernible mass (Figure 3). The spinal cord was unremarkable. Mild spondylosis deformans was present at L3-L4; however, there was no gross evidence of diskospondylitis. 

The entire brain, spinal cord, thoracic and abdominal viscera were fixed in 10% neutral buffered formalin, processed routinely, and embedded in paraffin wax. Sections (5 μm) were stained with hematoxylin and eosin (HE). Selected sections were subjected to immunohistochemistry (IHC) for detection of oligodendrocyte transcription factor 2 (Olig2; rabbit monoclonal antibody, 1:100 dilution, Abcam, Cambridge, MA, USA), glial fibrillary acidic protein (GFAP; pre-diluted rabbit polyclonal antibody, Dako, Carpinteria, CA, USA), ionized calcium-binding adapter molecule 1 (Iba-1; rabbit polyclonal antibody, 1:300 dilution, Biocare Medical, Concord, CA, USA), cluster of differentiation 3 (CD3; mouse monoclonal antibody, 1:25 dilution, Dako, Carpinteria, CA, USA), and paired box 5 (Pax5; mouse monoclonal antibody, 1:15 dilution, Dako, Carpinteria, CA, USA) using an automated system (Autostainer Link, Dako, Carpinteria, CA, USA) with a polymer-based horseradish peroxidase-conjugated detection kit with 3, 3′ diaminobenzidine tetrahydrochloride as the chromogen (EnVision™ FLEX high-pH kit, Dako, Carpinteria, CA, USA). Antigen retrieval was performed by heating in citrate buffer and endogenous peroxidase activity was quenched via immersion in a phosphate buffer (EnVision™ FLEX Peroxidase-blocking Reagent, Dako, Carpinteria, CA, USA). Slides were counterstained with hematoxylin. Sections of normal canine brain were used as positive controls for Olig2 and GFAP; sections of normal canine lymph node were used as positive controls for Iba-1, CD3, and Pax5. For negative reagent controls, primary antibodies were replaced with equivalent non-homologous immune sera. Negative internal tissue controls in immunolabeled slides consisted of non-glial, non-lymphoid, and non-histiocytic cells such as vascular endothelium, which did not demonstrate immunoreactivity with any of the antibodies. 

**Figure 4:**
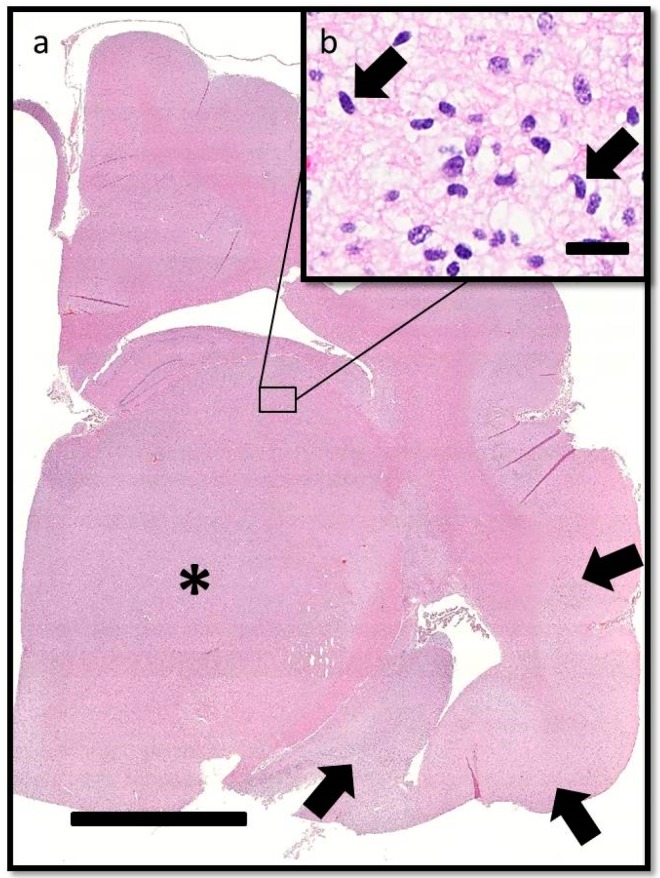
Transverse section of the brain at the level of the interthalamic adhesion. (**a**) Subgross photomicrograph showing loss of general anatomical detail within the thalamic nucleus and internal capsule due to a poorly delineated neoplastic mass (asterisk). Neoplastic cells also obscure the sylvian gyrus, hippocampus, and intervening parenchyma (arrows). HE. Bar = 5 mm. (**b**) Inset of (**a**). The neoplastic cells have indistinct cytoplasm, ovoid to elongated or curved hyperchromatic nuclei, and indistinct nucleoli (arrows). HE. Bar = 20 μm.

**Figure 5:**
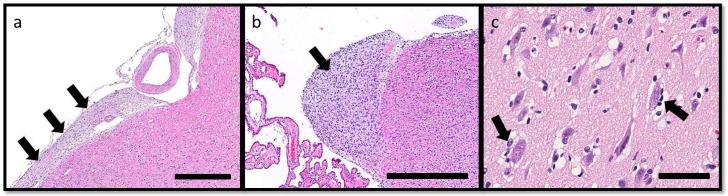
Secondary structures of Scherer, demonstrating migration of glioma cells through the brain, include: (**a**) subpial spread of neoplastic cells (arrows) along the ventral aspect of the brainstem, HE. Bar = 500 μm; (**b**) subependymal aggregation (arrow), HE. Bar = 500 μm; and (**c**) minimal perineuronal satellitosis (arrows). HE. Bar = 50 μm.

On histopathologic examination, ventral grey and white matter of the cerebrum (including the olfactory nuclei, olfactory tracts, corpus striatum, lateral septal nuclei, medial septal nuclei, thalamic nuclei and hippocampus), cerebellum, and brain stem (predominantly affecting the olivary nuclei) were bilaterally but asymmetrically infiltrated and expanded by dense sheets of neoplastic cells that merged gradually into the adjacent parenchyma, resulting in indistinct margins between normal and abnormal tissue (Figure 4a). The neoplastic cells had indistinct cytoplasm, ovoid to elongated or curved hyperchromatic nuclei, and indistinct nucleoli (Figure 4b). Anisokaryosis was mild and there was one mitotic figure per 10 400× fields. Multifocally, the neoplastic cells formed secondary structures of Scherer, including foci of subpial spread (predominantly along the ventral aspect of the brainstem; Figure 5a), subependymal aggregates (Figure 5b) with multifocal extension into and expansion of the leptomeninges, and minimal perineuronal satellitosis (Figure 5c). The neoplastic cell population was negative for Olig2, GFAP, Iba1, CD3, and Pax5. Scattered among these cells were variable numbers of GFAP-positive astrocytes, Olig2-positive oligodendrocytes, and Iba1-positive microglia. All examined sections of spinal cord were histologically unremarkable. Based on the cellular morphology, immunolabeling characteristics, and anatomical location, a diagnosis of gliomatosis cerebri was made. 

## 3. Discussion

In the present case, gliomatosis cerebri was diagnosed in an eight-year-old, neutered, female, long-haired cat by magnetic resonance imaging, and histological and immunohistochemical examination. Clinical signs in this patient are consistent with the neuroanatomic location of the neoplasm. The mild proprioceptive ataxia was likely due to neoplastic infiltration of the cerebellum and brainstem (predominantly affecting the olivary nucleus). The cerebellum is responsible for coordinating motor function to maintain appropriate posture and movements. To accomplish this, it uses proprioceptive information to modulate the activity of the upper motor neuron (UMN) nuclei and motor cortex, which then influence lower motor neuron (LMN) activity [8]. Cells from the olivary nucleus contribute by extending into the cerebellum to aid in voluntary muscle movement [9]. An additional clinical sign in this patient was cervical spinal hyperesthesia, a common finding in dogs and cats with intracranial neoplasia [10].

MRI of the spinal cord revealed a linear T2 hyperintense, non-enhancing T1 hypointense area in the dorsal aspect of the C2-C5 cord with dilation of the central canal. These imaging characteristics are consistent with a syrinx. Although the entire spinal cord was evaluated grossly and histopathologically, the presence of a syrinx was not able to be confirmed. The reason for the discrepancy is unknown; however, it is possible that the lesion was more subtle than imaging suggested and that collapse occurred during the post-mortem interval. 

GC has been reported in the brains of people [1,11], dogs [12,13], and in a single cat [14]. According to the 2007 WHO classification, GC is considered to be a Grade III astrocytoma, a malignant neoplasm characterized by glial cells that invade adjacent normal tissue [3]. Data suggest that men are affected more commonly than women [2] and although it has been reported in all age groups, it is most common in the fifth decade of life [15]. As seen in this case, the T2-weighted MRI of the brain (in both people and dogs) usually shows vague, heterogeneous, non-contrast-enhancing hyperintensities resembling inflammatory disease, vasculitis, leukoencephalopathies, or venous infarcts [2,13,16]. GC MRI findings are generally considered to be characteristic of an astrocytoma, but with wider cerebral dissemination [17]. Human GC is typically characterized by widespread, diffuse, bilateral neoplastic infiltrates in at least three cerebral hemispheric lobes. There is also frequent involvement of the cerebellum, brainstem, and spinal cord [17]. Although imaging was not performed in the previously reported case of GC in the brain of a cat, histopathologic evaluation confirmed a diffuse neoplastic cell infiltrate in the cerebrum with minimal infiltrates in the metencephalon; neoplastic cells were not observed in the cerebellum or spinal cord [14].

GC in the brains of people and dogs, and in the single case report in a cat, is characterized histologically by infiltrates of cells with ovoid to slender nuclei and indistinct cytoplasm admixed with astrocytes, oligodendrocytes, and microglia. Typically, the infiltrating cells fail to form a grossly evident mass [12,14]. In people, the neoplastic cells are often positive for GFAP, although some fusiform cells are GFAP-negative [18]. In dogs, the neoplastic cells are positive for vimentin and nestin [19] and negative for pan-cytokeratin, neuron specific enolase (NSE), neurofilament, CD133, S100, GFAP, CD18, CD3, CD45RA, CD79α, and B lymphocyte antigen 36 (BLA.36) [12,19]. GC has been reported in the feline spinal cord, with a similar hyperintense T2-weighted MRI signal as described in the brains of people and dogs. Feline brain and spinal cord GC has been characterized by neoplastic infiltrates that are GFAP-negative [14,20,21]. In feline spinal cord GC, the neoplastic cells are also reported to be negative for Ki-67, Pax5, and CD45, with strong cytoplasmic immunoreactivity for BLA.36 [21]. Additional microscopic features of GC in people and dogs include perineuronal and perivascular satellitosis, subpial and subependymal neoplastic aggregates, and migration of neoplastic cells within white matter tracts (intrafascicular spread) [9]. These patterns of glioma cell infiltration are referred to as the secondary structures of Scherer; they were not observed in the previously reported case of GC in the feline brain [14,22].

Histopathologic differentials for gliomatosis cerebri include other diffusely infiltrating astrocytomas (diffuse astrocytoma, anaplastic astrocytoma, and glioblastoma), oligodendroglial tumors, mixed gliomas, and lymphoma. Diffuse astrocytomas typically arise supratentorially in the frontal and temporal lobes [17]. Similar to the neoplasm in this patient, diffuse astrocytomas usually have a lack of necrosis, foci of microvascular proliferation, and secondary structures of Scherer [23]. Unlike the neoplasm in this patient, however, diffuse astrocytomas are composed of fairly uniform infiltrating fibrillary astrocytes that have elongated, enlarged hyperchromatic nuclei, scant cytoplasm, and strong GFAP and vimentin cytoplasmic immunoreactivity [17]. Anaplastic astrocytomas typically arise from the same locations as diffuse astrocytomas; however, they have greater overall cellularity and neoplastic cell density. Additionally, unlike diffuse astrocytomas, cells of anaplastic astrocytomas have minimal cytoplasm with nuclear atypia, a greater mitotic index, and higher proliferative activity. The neoplastic cells of both anaplastic and diffuse astrocytomas generally have strong GFAP or vimentin immunoreactivity. Glioblastoma often occurs in the frontal and temporal lobes. Grossly, there are typically well-delineated areas of necrosis and hemorrhage. Histologically, these neoplasms are hypercellular and pleomorphic with anisokaryosis, numerous mitotic figures (some of which are abnormal), intratumoral microvascular proliferation, pseudopalisades of glial cells, and foci of necrosis. In the dog, glioblastomas typically have strong cytoplasmic GFAP immunoreactivity, like other astrocytomas. [17]. Based on cellular morphology, immunolabeling characteristics (lack of GFAP immunoreactivity), and a lack of secondary tumoral characteristics (such as microvascular proliferation), the neoplasm in the present report is not consistent with the above-mentioned astrocytoma subtypes. 

As mentioned, oligodendroglial tumors, mixed gliomas, and lymphoma were additional possible histopathologic differentials in this case. Oligodendrogliomas often occur in the frontal, parietal, and temporal lobes; meningeal invasion/metastasis and dissemination within the ventricular system can also occur. In people, dogs, and cats, these tumors are usually well-demarcated and have a grey gelatinous consistency [17,24,25]. Histologically, oligodendrogliomas are characterized by densely packed, fairly uniform cells with round nuclei and well-delineated cytoplasmic borders. Multifocally, there are typically lakes of myxoid material and thin-walled branching capillaries that produce a characteristic “chicken-wire” appearance. Grade III oligodendrogliomas have increased nuclear atypia, foci of necrosis, thrombosis, hemorrhage, and prominent microvascular proliferation. Neoplastic oligodendroglial cells have nuclear Olig2 immunoreactivity and lack GFAP immunoreactivity [17]. Mixed gliomas have astrocytic and oligodendroglial components and are therefore known as oligoastrocytomas. Both cell populations typically have strong cytoplasmic GFAP immunoreactivity, minimal pleomorphism, and few mitoses (unless it is an anaplastic variant). Anaplastic variants, in addition to having anisocytosis, anisokaryosis, and increased numbers of mitotic figures, also have foci of microvascular proliferation [17]. CNS lymphoma can be primary, intravascular, or metastatic. Primary CNS lymphoma often forms a single mass lesion and is composed histologically of sheets of pleomorphic round cells which extend from perivascular aggregates [17,26]. Metastatic lymphomas are typically confined to the leptomeninges and dura, with foci of parenchymal invasion. CD3 and Pax5 immunolabeling can help determine whether these lymphomas are of T-cell or B-cell origin, respectively. [17]. Cellular morphology, lack of immunoreactivity for GFAP, CD3 and Pax5, and lack of secondary tumoral characteristics (such as microvascular proliferation) preclude the diagnosis of an oligodendroglial tumor, mixed glioma, or lymphoma in this patient.

## 4. Conclusions 

Based on MRI findings, gross examination, anatomic location and behavior, neoplastic cellular morphology, and immunolabeling characteristics, this patient was diagnosed with GC of the brain. Although GC has previously been reported in the brain of a cat, this current case report is unique because it describes MRI findings, details histopathologic findings, and characterizes neoplastic cellular immunoreactivity. Although uncommon, gliomatosis cerebri should be considered as a differential diagnosis for brain neoplasia in the cat.

## Abbreviations

The following abbreviations are used in this manuscript:

GC: Gliomatosis cerebri
MRI: Magnetic Resonance Imaging
